# Physics of the Mind

**DOI:** 10.3389/fnsys.2016.00084

**Published:** 2016-11-15

**Authors:** Leonid I. Perlovsky

**Affiliations:** ^1^MGH/HST Martinos Center for Biomedical Imaging, Medical School, Harvard UniversityCambridge, MA, USA; ^2^Psychology and Engineering Departments, Northeastern UniversityBoston, MA, USA

**Keywords:** physics of the mind, neuroscience, cognition, dynamic logic, knowledge instinct, aesthetic emotions, consciousness, beautiful

## Abstract

Is it possible to turn psychology into “hard science”? Physics of the mind follows the fundamental methodology of physics in all areas where physics have been developed. What is common among Newtonian mechanics, statistical physics, quantum physics, thermodynamics, theory of relativity, astrophysics… and a theory of superstrings? The common among all areas of physics is a methodology of physics discussed in the first few lines of the paper. Is physics of the mind possible? Is it possible to describe the mind based on the few first principles as physics does? The mind with its variabilities and uncertainties, the mind from perception and elementary cognition to emotions and abstract ideas, to high cognition. Is it possible to turn psychology and neuroscience into “hard” sciences? The paper discusses established first principles of the mind, their mathematical formulations, and a mathematical model of the mind derived from these first principles, mechanisms of concepts, emotions, instincts, behavior, language, cognition, intuitions, conscious and unconscious, abilities for symbols, functions of the beautiful and musical emotions in cognition and evolution. Some of the theoretical predictions have been experimentally confirmed. This research won national and international awards. In addition to summarizing existing results the paper describes new development theoretical and experimental. The paper discusses unsolved theoretical problems as well as experimental challenges for future research.

## What is physics of the mind?

The common to all areas of physics is a methodology that first, concentrates on finding few fundamental laws and their mathematical formulations; second, a mathematical theory developed from these few “first principles” that explains a vast area of knowledge without contradicting known facts; third, makes unexpected theoretical predictions, which could be verified experimentally, and actual experimental verifications which confirm or disconfirm the theory.

This paper briefly summarizes previously developed aspects of the physical theory of the mind and presents new developments. It discusses the first principles identified by a number of leading neuroscientists, mathematical methods suitable for their modeling, perception and cognition mechanisms based on these principles, the mechanisms of an approximate mental hierarchy. The physics of the mind and the related mathematical theory are extended toward the dual hierarchy of interactions between cognition and language, high cognition including emotions of the beautiful, a controversial idea of the meaning of life, as well as functions of these high principles in cognition. It is further extended toward emotional prosody of speech as well as cognitive functions and the reasons for evolution of musical emotions from animal cries to Bach and Justin Bieber.

This theory does not contradict existing knowledge, explains psychological facts that have been poorly understood previously, and has made a number of unexpected experimentally verifiable predictions. The paper discusses theoretical predictions that have been experimentally confirmed (or tentatively confirmed). Among these confirmed predictions are mechanisms of perception and cognition, mathematical model of the mind that overcomes computational complexity, interaction between cognition and language, the nature and mechanisms of the beautiful and the meaning of life, as well as the cognitive functions and reasons for origin and evolution of musical emotions. Computational complexity interfered with modeling the mind, artificial intelligence and machine learning since the 1960s, a mathematical theory overcoming this difficulty is described. The paper presents physics of the mind that mathematically models psychological mechanisms at the functional level. Self-organization processes are related to thermodynamics and informational theories. The functional theory has been partly related to neural mechanisms, and some of these relations have been experimentally confirmed. The paper discusses predictions, which open vast areas for future research.

## Fundamental principles of the mind-brain

This section describes several fundamental principles of the mind-brain, later the paper describes mathematical models for some of them.

*Concepts* are a mechanism of understanding objects, events, and abstract ideas. Their contents are stored in neural representations. In the processes of perception concepts project their contents to the visual cortex to match sensory projections. Concepts are also called mental models of events and objects. In a simple case a concept is memory. The analogy with models is literal and neural representations are also called mental models. Mathematical models of concept mechanisms is discussed in (Perlovsky et al., 1997, 2011; Perlovsky, 2006a). Proof of detailed theoretical predictions of the mechanism in experimental neuro-imaging, including detailed descriptions of the brain regions involved was obtained by Bar et al. (2006) and Kveraga et al. (2007). The moment of perception is a match of these images.

*Instincts* are ancient mechanisms of survival. This paper follows Grossberg and Levine (1987) theory, which has been modeled mathematically and is appropriate for developing physics of the mind. This theory considers the instinct mechanism resembling “neural sensors that measure vital parameters important for functioning and survival” (Grossberg and Levine, 1987; Perlovsky, 2006a). For example, a low blood glucose level specifies an instinctual need for food. Measurements of glucose level sensors and the requirement to keep glucose level within bounds is a mechanism of instinct.

*Emotions* designate various mechanisms which are surveyed in a number of publications. Following Grossberg and Levine (1987) theory of drives and emotions the mechanism of emotions are neural signals connecting instinctual and conceptual brain regions. Emotions, emotional neural signals, related states and feelings communicate instinctual needs to conceptual recognition-understanding mechanisms. Their function is to motivate behavioral and conceptual representation-models, which correspond to objects or events that can potentially satisfy instinctual needs, so that these models receive preferential attention and processing resources within the brain. Thus emotions evaluate concepts for the purpose of instinct satisfaction. Emotional signals and related states of the mind are felt as emotional feelings.

Psychological research of emotions is usually limited to basic emotions, which are related to satisfaction of bodily instinctual needs, named by specific words, and limited in number to a few different emotions. There are only few basic emotions; they are a small part of our emotional abilities, the most ancient and noticeable ones. Our higher cognitive abilities involve many “continuous” emotions, which include aesthetic emotions discussed later, related to knowledge, including processes of learning, emotions in the voice prosody, emotions of cognitive dissonances, as well as musical emotions described later.

*Behaviour* is governed by several mechanisms. The most interesting for the initial development of physics of the mind is the mechanism of behavioral concepts; it is similar to the mechanism of cognitive concepts discussed above with the difference that behavioral concepts govern behavior. Most of human behavior occurs in the mind, it is directed at improving concepts, understanding, and knowledge.

*Cognitive hierarchy* is an approximately hierarchical structure (Kosslyn, 1980; Grossberg, 1988) of mental models and aesthetic emotions (discussed later) extending from sensory-motor representations at the bottom of the hierarchy, higher up to concepts of objects, contexts, situations, and many levels of abstract concepts, to the top of the hierarchy, which content will be elucidated in the paper. This description is not quite accurate, especially for neural mechanisms below objects; one can look for details e.g., in Grossberg publications, but it is adequate for higher levels. The hierarchy is functional, it is not organized from the bottom to the top along a specific geometric axis. Processes of understanding involve interactions among models at lower and higher levels. In these interactions higher level models are improved for better correspondence to lower level models; a higher level model unify lower level ones for creating a more abstract and general concept. The interaction is two-way: lower level models are also improved for better match to the details of the situation (lower level models) and for better matching the top level one. Neural signals involved in these interactions are called bottom-up, BU, and top-down, TD, signals.

*The knowledge instinct*, KI, is a special instinctual mechanism related to knowledge acquisition and improvement of concept-models (Perlovsky, 2001, 2006a, 2007b, 2008b; Perlovsky et al., 2011). Its model is an extension of Grossberg and Levine (1987) theory of bodily instincts to cognition. KI is similar to other instincts, it involves sensor-like neural mechanisms that measures similarities between patterns in sensor data and mental models, or more generally between BU and TD signals. As discussed later, in humans and other higher animals mental models are vague, and matching them to objects and situations requires adapting them to BU signals. KI drives this adaptation. No perception or cognition would be possible without KI. For this reason KI is a most important instinct. KI is not related to bodily needs but to “higher” needs for cognition and in this sense it can be termed a higher instinct.

*Aesthetic emotions* are related to satisfaction of KI and they are modeled mathematically by changes in KI. This theoretical prediction have been experimentally confirmed (Perlovsky et al., 2010; Schoeller and Perlovsky, 2015, 2016). Relation of aesthetic emotions to knowledge was established by Kant (1790), although he could not formulate his thoughts with mathematical precision, the adequate mathematics did not exist at the time. His thoughts have not been understood by his followers. Aesthetic emotions are related to learning and understanding at every level of the mental hierarchy, understanding is pleasant (Perlovsky, 2001, 2006a; Perlovsky et al., 2011; Schoeller and Perlovsky, 2015, 2016). But we do not relate understanding at lower levels to the beautiful. Later this paper connects aesthetic emotions to the emotions of the beautiful.

*Perception* of objects refers to recognition of more or less familiar objects, and sometimes to noticing unfamiliar objects. Visual perception involves neural projections of retinal images to the visual cortex (BU). At the same time existing models (of expected objects) project TD signals to the visual cortex (TD). Driven by KI (or in other words, motivated by aesthetic emotions) TD and BU projections of models on the visual cortex are modified to match each other. When match is successful, perception occurs (Grossberg, 1988). This process is modeled mathematically (Perlovsky et al., 2011), and detailed predictions of this model are experimentally confirmed (Bar et al., 2006; Kveraga et al., 2007).

The above principles describe self-organization of conceptual and emotional mechanisms of perception and cognition. They encompass the mechanisms of imagination, intuition, planning, conscious, unconscious, and others, including higher abilities and aesthetic emotions (Perlovsky, 2001, 2006a, 2010d; Perlovsky et al., 2011). Most brain operations are unconscious, for example, individual neuronal firings usually can never be accessed by consciousness. This paper refers to the brain-mind neural processes that are not accessible to consciousness as being unconscious, and there are various degrees of unconsciousness. Some processes could never become conscious; others can be accessed by consciousness with significant mental effort, as in creative processes; still others become conscious under changing circumstances without special effort. Many theoretical predictions have been confirmed in experiments (Kosslyn, 1980; Grossberg, 1988; Perlovsky et al., 2010; Schoeller and Perlovsky, 2015, 2016).

*Vague Representations.* Mental models are not crisp like visual perceptions. A simple experiment can prove this. Look at an object in front of you and then recollect this object with closed eyes. This visual imagination is vague, one cannot recollect even a simple everyday object in all details with closed eyes. Imagination is a TD neural projection from memory to the visual cortex. Vagueness of imaginations is a consequence of vagueness of mental models (Perlovsky, 2016b).

This property of models is fundamental for perception. The reason is that an object would never appear exactly same as during previous perceptions; angles, lightings, surrounding objects would always be different. Therefore previously remembered objects would not match new object projections from retina to visual cortex. Attempts in artificial intelligence (AI) to recognize objects by matching sensory images to previous images took many years and resulted in failures. The number of possible modifications of previous images to match a new image, are combinatorially large. This number is larger than all interactions of all elementary particles in the Universe, therefore the resulting complexity is unsolvable. The problem is called combinatorial complexity, CC (Perlovsky, 1998). Vague models avoid a need to consider combinations. The vague-to-crisp process is fundamental for self-organization, perception and cognition; vague representations and processes are not conscious, possibly for this reason vagueness of representations has not been appreciated by psychologists and mathematicians modeling the mind, and this is the reason why mind processes have not been mathematically modeled and understood in artificial intelligence (Perlovsky, 2001; Russell and Norvig, 2010).

*Dynamic logic*, DL, is a mathematical technique modeling the brain-mind mechanism of matching vague models to crisp projections from the retina (Perlovsky, 2001, 2006a, 2013c; Perlovsky et al., 2011). Adequacy of DL vague-to-crisp process has been experimentally proven in (Bar et al., 2006; Kveraga et al., 2007). M. Bar and colleagues proved that the initial state of models is vague. The process “from vague to crisp” until models match retinal projections take approximately 150 ms. These includes many neuronal operations: about 10 ms per firing of a neuron, while tens of thousands of neurons are participating in parallel. The initial part of this process cannot be accessed by consciousness, vague models and processes are not accessible to consciousness. Conscious perceptions occur only at the moment of model-projections matching object-projections from the retina.

DL is a process-logic, it avoids logical states until the very end of the DL-process (Perlovsky, 2006a, 2013c). This is essential because CC has been shown to be equivalent to Gödelian incompleteness of logic when applied to finite systems, such as computers or brains (Perlovsky, 2013d). It is interesting to note that the founder of logic, Aristotle explained to his students that logic is needed to argue what has been already understood, but not for understanding of new phenomena, and logic should not be used for understanding working of the mind. Aristotelian theory of the mind is similar to DL, the mind understand the world by using forms that today we call models or representations. Initial states of the forms are potentialities, which are not logical. In the process of “the mind meeting matter”, which today we call interactions of TD and BU signals, forms become actualities, which are logical states.

This section summarizes several fundamental principles of the mind: instincts, emotions, concepts, cognitive hierarchy, the knowledge instinct, aesthetic emotions, perceptions, vague model-representations, dynamic logic. Not all of these principles are independent, e.g., KI and aesthetic emotions are extensions of general principles of instincts and emotions, vague representations are a part of dynamic logic; this repetition is justified by importance of the correspondence principles. Perception is a mechanism explained from fundamental principles. Mathematical foundations of these principles have been discussed, mathematical details will be presented later as well as few other fundamental principles. Identification of few fundamental principles is a first step toward developing physics of the mind.

## The beautiful and meaning of life

**Figure 1:**
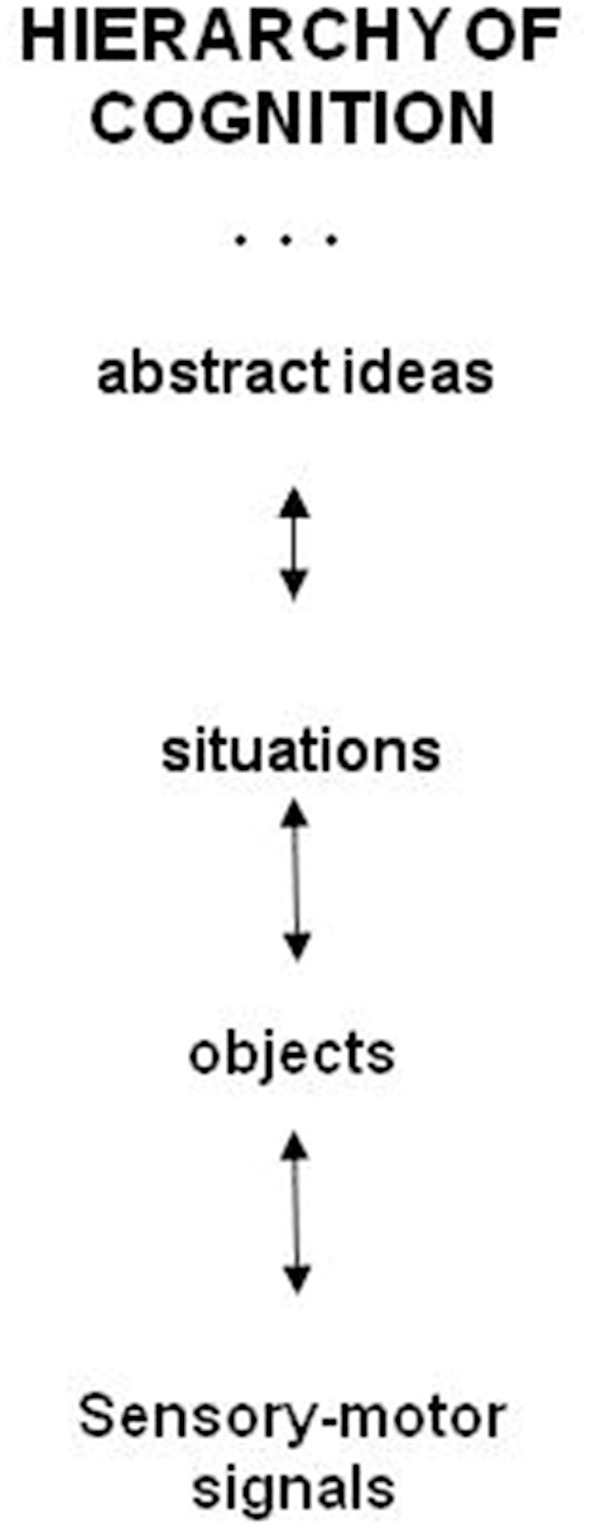
**The hierarchy of cognition**. Cognition is organized into an approximate hierarchy of concepts from objects to abstract concepts and higher up to the highest cognitive concepts of the meaning and purpose. Aesthetic emotions function as motivators of learning concepts at every level, at the top of the hierarchy these are the emotions of the beautiful.

The mind mechanisms are organized into an approximate hierarchy of concepts and aesthetic emotions. Cognitive hierarchy is illustrated in Figure 1.

The hierarchical organization of cognition and related brain structures are reviewed in (Badre, 2008). The hierarchy evolved for the purpose of developing more abstract and general concept-models (Perlovsky, 2006a). Consider a perception-cognition process of an everyday situation, e.g., a professor office. The knowledge instinct first drives the mind to perceive and understand objects in the office: chairs, computers desks, shelves, books…Animals also understand individual objects. Next, the knowledge instinct drives us to understand the concept “office” as a unity of objects. A mathematical model of this process was developed in (Ilin and Perlovsky, 2010; Perlovsky and Ilin, 2010a,b). The higher level abstract concepts we understand due to corresponding concept-models, such as “office.” Similarly, we understand a “concert hall,” and other situations by using higher-level concepts that our mind evolved for this purpose.

I will repeat the word purpose; every higher-level concept and its mechanisms evolved in individual learning as well as in genetic and cultural evolution with a purpose to be able to transform many lower-level concepts into a unified meaning. In this understanding lower-level concept-models acquire higher-level meanings and develop a more abstract understanding than lower-level meanings. This way our understanding of the surrounding world evolves from a “book” to an “office,” to a “university”, to an “educational system,” and so on…to models near the top of our mental hierarchy. These “top” models “attempt to make sense, to understand the meaning of our entire experience. We understand-perceive-feel them as related to the meaning and purpose of our lives” (Perlovsky, 2006a, 2010c).

Models at every level unify lower level models, for example a situation-model symphony hall unifies lower level object-models: chairs, listeners, scene, etc. Continuing this argument to the top of the hierarchy, one concludes that models at the top unify the entire life experience. These top representations are understood as the meaning and purpose of life. As discussed, even lower level concepts are vague. Abstract concepts built on top of many levels of vague models are even vaguer (Perlovsky, 2011c), therefore the meaning and purpose of life are not finite exactly defined ideas like objects perceived with opened eyes. The next section discusses why sometimes it may seem that we can crisply formulate these ideas.

Learning of models at every level is driven by KI operated at that level, or in other words is motivated by aesthetic emotions at this level. At lower levels aesthetic emotions could be below consciousness. At the top of the hierarchy the highest aesthetic emotions are emotions of the beautiful, sometimes these emotions could be strong and even produce physiological effects, aesthetic chills (Perlovsky, 2002, 2006a, 2007c, 2008a, 2010b,c; Mayorga and Perlovsky, 2008; Schoeller and Perlovsky, 2015, 2016).

Let me emphasize that defining emotions of the beautiful as the highest aesthetic emotions (that is aesthetic emotions near the top of the mental hierarchy) corresponds to a well-accepted human intuition. Kant (1790) has been the first who related beautiful to the meaning and purpose of human life, yet his intuitions have been well ahead of understandings of his contemporaries. The only widely known Kantian idea about the beautiful is that it is “aimless purposiveness,” often with emphasis on aimless, because the purposiveness of the beautiful is not understood even in contemporar y aesthetics. This is clearly seen when visiting museums of contemporary art.

While progress in understanding of the purpose of human life can be seen in art evolution from cave art to the 19th century, in the 20th century art the exploration of purposiveness has been disappearing. In rare pieces of contemporary art the meaning and purpose of life is explored. This ignoring Kantian intuitions in contemporary art is likely to be closely related to the fact that the idea of “science” become important in cultural life (without understanding of what science is). Existing science does not understand what is beautiful. For example, G. Dickie, an influential philosopher of art, a president of the American Society for Aesthetics, and author of popular textbooks developed an “institutional theory of aesthetics” which defines beautiful as what has been accepted as beautiful by respected art institutions; it is still widely accepted as a state of the art in understanding of the beauty. In wide culture beautiful is understood as more related to sex than to the meaning of life. In university courses on aesthetics beauty is related to shapes, colors, forms, and progressive social uses of art, rather than to the purposiveness of life. So, I would again emphasize that the theoretically predicted properties of the emotions of the beautiful, their relations to the meaning of life are unexpected in contemporary aesthetics and contradictory to accepted views. Nevertheless these theoretical predictions have been experimentally tested and confirmed (Schoeller and Perlovsky, 2015, 2016), which is the fundamental property of the science of physics of the mind.

This section gives an example of complicated cognitive mechanisms explained from the first principles, and making theoretical predictions that have been tested in experiments.

## The dual hierarchy, language, and cognition

The recognition that language and cognition are not the same, that these abilities are served by different mechanisms of the mind, began a revolution in 20th century linguistics initiated by N. Chomsky (1957). Many psycholinguists and evolutionary linguists today disagree with Chomsky's complete separation of language from cognition (Cangelosi and Parisi, 2002; Christiansen and Kirby, 2003; Steels, 2011), yet many questions remained unanswered. What is the difference between cognition and language? Language is so important for thinking that it is difficult to comprehend what cognition would be without language. How does cognition interact with language? Do we think with words, or only use words as labels when a chunk of a thinking process is complete? There is virtually infinite number of possible associations between words and objects, so how is it possible that every child learns correct associations? Why children learn language by the age of 5 or 7, but do not think like adults until much later? What exactly are the changes in neural mechanisms? Do adults really understand what they say, and what does it mean to really understand? Some people are good at speaking language, while not equally good in discussing with other people, or understanding the real world. Opposite examples could be found. The science needs to understand the mechanisms of language and cognition interactions; why they are so interdependent, and so separate? What neural mechanisms animals need to learn language?

These questions and many more can be explored with adding one fundamental principle to those previously discussed. *Dual model* (Perlovsky, 2004, 2006a, 2007a,b, 2009a, 2013b) is a fundamental principle of the mind modeling interaction between language and cognition. According to the dual model, every mental model has a cognitive and language parts. Their initial states are vague. In a newborn brain most of cognitive and language models are placeholders without specific contents.

**Figure 2:**
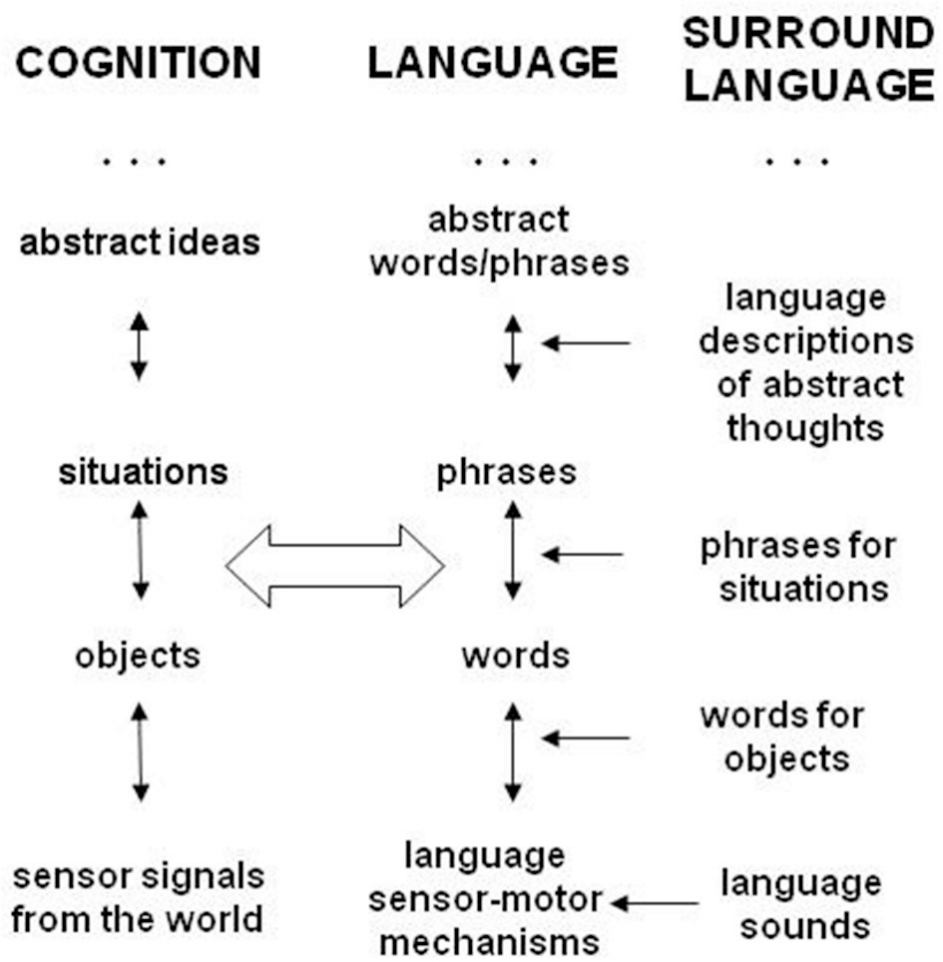
**The dual hierarchy model (see Perlovsky, 2006a)**. Language and cognition are organized into approximate dual hierarchy. Every representation has two parts, cognitive and language. Learning language is grounded in the surrounding language throughout the hierarchy. Cognitive hierarchy is grounded in experience only at the very “bottom”; the cognitive hierarchy is constructed from experience guided by language.

Adding dual models to the cognitive hierarchy in Figure 1 leads to two parallel hierarchies of language and cognition shown in Figure 2 (Perlovsky, 2006a, 2009a, 2013b). In childhood language representations are learned fast and become crisp and conscious. This is possible because language acquisition relies on language spoken around, in which contents of language models, words, phrases, are “ready-made” for learning. But many cognitive models remain vague until much later; cognitive learning is much more difficult, because cognitive models do not exist in the world “ready for learning.” At an early age, everyone can talk about good guys and bad guys, but nobody even at 40 or 80, can use these concepts without errors in real life. Ideas of good and evil have been discussed for millennia.

Throughout the hierarchy, linguistic parts of representations are crisp and conscious in every mind at an early age, but equally crisp and conscious cognitive models my never be understood. Representations of objects are acquired early, alongside with language, because we see objects ready-made for cognitive learning. But contents of abstract concepts do not exist in the world “ready-made.” Not every combination of objects or events is worth learning as a separate abstract concept, only few combinations are important for understanding. Therefore learning abstract concepts require experience guided by language. If a concept does not exist in language, it is likely that it does not exist in cognition, and corresponding events are not even noticed. Many people speak words without full cognitive understanding of what these words designate in real life. These aspects of language-cognition have not been duly noticed or explained; the mechanism of the dual model explains them. Language models refer to facts of language, and not directly to events in the world. Cognitive models combine language with experience and refer to events in the world.

The dual model hypothesis is tentatively supported by experimental data (Franklin et al., 2008). These authors describe that certain representations based in the right brain hemisphere in pre-linguistic infants are rewired to the left hemisphere as language is acquired. Brain modules and neural connections involved in the dual models and knowledge instinct were discussed in (Levine and Perlovsky, 2008a,b).

The dual model has been fundamentally important for the emergence of the hierarchy of the mind. Learning should be grounded in experience (Cangelosi and Riga, 2006; Tikhanoff et al., 2006; Coventry et al., 2010). But concept-models of cognition are grounded in experience only at the lower levels of concrete objects; at this level human abilities are not much different from that of pre-human animals' (Spelke and Kinzler, 2007). Understanding situations and abstract concepts cannot be based on experience alone. The referenced publications discuss in detail why this is mathematically impossible: there are simply too many combinations of objects and events (more then all elementary interactions in the life of the Universe). No life's experience would ever be sufficient to learn which combinations are important for noticing them and learning as separate abstract concepts.

The dual hierarchy model offers a resolution of an old problem of sign and symbol (Perlovsky, 2007a). “Symbol is the most misused word in our culture” (Deacon, 1989). “Symbol” is used in simple cases referring to traffic signs, or axiomatic mathematical notations, and in the most profound cases of cultural and religious symbols. The dual model explains that a sign corresponds to a language part of the dual model (even if it is a part of a special sign system, such as notations in chess or mathematics). Symbols can be used profoundly to denote processes of sign interpretation, connecting language and cognitive parts of the model in DL processes from vague to crisp.

The dual model answers questions formulated at the beginning of this section; it explains how cognition interacts with language: a cognitive process proceeds using both cognitive and language representations. This enables thinking to be grounded in the real world to the extent available to the thinker, and still proceed using language whenever understanding of the real world is insufficient. Similarly both representations are used when speaking, depending on one's abilities and preferences; language or cognitive models receive preference in the speaker's mind. People of “speaking type” can shift between cognitive and language models automatically and without notice, while “cognitive types” may concentrate on cognition.

Associations between words and objects or events are learned among virtual infinity of possible association by every child through the process “from vague to crisp” modeled by DL. The reason language is learned first and cognition is learned later because language representations exist “ready for learning” in the surrounding language, while learning cognitive representations requires experience and guidance by language. Adults are different from children in that a larger percentage of their cognitive models are crisp. Still a significant part of what most people are saying is understood only in terms of language, but not necessarily in terms of real world entities. Animals cannot learn language no cognitive hierarchy because they are missing a neural mechanism of the dual language-cognitive model.

To summarize, cognitive models at higher levels are learned based on both, life experience and language models. In this process language guides cognition: language identifies for cognition, which combinations of lower level concepts are meaningful for learning as a higher level concept. Language hierarchy is learned “ready-made” from the surrounding language at an early age. During the rest of an individual's life the knowledge instinct drives the mind to learn the cognitive hierarchy from life experience in correspondence with the language hierarchy. If a certain idea does not exist in a language, this idea does not exist in cognition, and corresponding events would not even be noticed. Cognitive models are grounded in language. Many theoretical predictions of the dual model hierarchy in this section have not yet been experimentally proven, they should be considered as hypotheses and their experimental verifications are topics for future research.

This section, as the previous one, gives an example of a vast field of complicated cognitive mechanisms explained from the first principles, and making theoretical predictions that can be tested in experiments; few of these predictions have been tested experimentally. It answers many questions that could not even have been formulated previously. Detailed mathematical models are discussed in given references. The theory discussed in this section is a part of physics of the mind, it is a step toward making psychology a “hard” science.

## Emotionality of languages and cultures

In non-human animals voice muscles are controlled from ancient involuntary emotional centers. For this reason animal voicing is mostly inseparable from emotions. “Voluntary control of vocalization is limited” (Deacon, 1989; Schulz et al., 2005; Perlovsky, 2009b; Simonyan and Horwitz, 2011). Evolution of language, semantically loaded voice, required to free voice mechanisms from involuntary emotional control. For this purpose in the course of language evolution human brain evolve recent laryngeal control centers in cortex, which make possible voluntary control of voice muscles.

Involuntary emotionality of voice has been significantly reduced. With evolution of language an ability for strongly emotional voice has mostly evolved into a separate ability for song and music (Perlovsky, 2012c). But unconscious emotionality of voice could not completely disappear. This everyday low emotional prosody performs a highly important cognitive function: it motivates connecting sounds of words with their cognitive meanings (Perlovsky, 2011b, 2012a). Let me emphasize this possibly non-obvious point. Language and its main way of functioning, speech, can only function if sounds of words are perceived emotionally. If a word sounds produce no emotions and no motivations, this word has no meaning. I dwell on this point because it contradicts accepted understanding. “Emotional speech” often is used as a synonym of meaningless or at least devoid of deep meaning, which could be true, especially if emotionality is high and emotions overtake the reason. Here I emphasize the opposite point: no emotionality indicates absence of any interest, and therefore convey no meaning. Proper emotionality is essential (Perlovsky, 2009b, 2011a). Low emotional prosody, which is below the level of consciousness has not been studied. This is an important area for future research.

The following part of this section makes theoretical predictions that follow from the few basic principles formulated above, mathematical models have been presented in given references, and the theoretical predictions are experimentally testable and will be tested in the near future. Emotional prosody of human voice, even if unnoticed, affects the entire psyche and even culture. In pre-human animals conceptual and emotional systems (understanding and evaluation) are less differentiated than in humans. Animal cries engage their psyche as a whole, rather than conceptual and emotional mechanisms separately. For example consider calls of vervet monkeys (Seyfarth and Cheney, 2003). The calls designate types of predators around; still “understanding of a situation (concept of danger), evaluation (emotion of fear), and behavior (cry and jump on a tree) are not differentiated, each call is a part of a single concept-emotion-behavior-vocalization psychic state with very little differentiated voluntary control” (Perlovsky, 2006a).

Humans on the opposite have separate mechanisms of emotions, concepts, and behavior. Differentiation of psychological states with voluntary control over each part must have evolved contemporaneously with evolution of language and rewiring of the brain.

It follows that language, while contributing to developing detailed ability for concepts, also contributed to separating and perfecting functions of concepts, emotions, and behavior. This differentiation destroyed the unity of psyche inherited from the pre-human past. Language evolution also led to losing unity of psyche, started losing wholeness. While in pre-human animals every element of knowledge is tightly connected to emotional evaluation of a situation, and to appropriate behavior satisfying instinctual needs, this is not so for humans. A significant part of cultural knowledge formulated in language is not emotionally connected to human instinctual needs. This is tremendously advantageous for development of conceptual culture, for science, and technology. Humans can deliberately discuss ideas.

But this freedom of deliberate conversation and clear conceptual thinking exerts a price on human psyche. Human psyche is not necessarily unified. Language is not directly linked to instinctual mechanisms. Often knowledge developed in culture does not fit with instinctual requirements that remain our inseparable part. In addition some elements of knowledge often contradict other elements. Human psyche must be unified by the highest models of the meaning and purpose evolved for this purpose at the top of the hierarchy of the mind (Perlovsky, 2007b, 2009b, 2013b). Therefore contradictions in the system of knowledge, a disconnect between knowledge and instincts, the lost synthesis, lead to internal crises and may cause clinical depressions. When psychic states missing synthesis preoccupy majority of population, knowledge loses its value, including knowledge of social organization, cultural calamities occur, wars and destructions (Diamond, 1997; Perlovsky, 2006a, 2007b, 2012d). Evolution of culture requires a balance between differentiation and synthesis. Differentiation is the very essence of cultural evolution. But it may lead to emotional disconnect between conceptual knowledge and instinctual needs, to the lost feeling of the meaning and purpose, including the purpose of any cultural knowledge, and to cultural destruction.

There is much evidence that languages differ in their emotional and conceptual contents (Guttfreund, 1990; Buchanan et al., 2000; Harris et al., 2003; Perlovsky, 2007a,b, 2009b, 2012d). While all contemporary languages lost involuntary connections between sounds and emotions characterizing animal vocalizations, this “hardwired” connections between voice and emotions has been replaced by habitual connections. As long as sounds of a language remain unchanged, the language maintains historical connections between word sounds and associated emotions. But if sounds of a language change fast, this historical connections might be lost.

A significant mechanism affecting a speed of language sound change and therefore emotionality of the language is word morphology, such as inflections expressing grammatical cases, voices, aspects, genders, numbers, tenses, and other constructs. A strongly inflected language may have dozens or even many dozens of inflections expressed by affixes and other grammatical devices. Every child hears these affixes every day, therefore knows how to pronounce them, even if does not know which grammatical category it expresses and when it should be used. In inflectional languages, like most European languages, pronunciation of affixes is to some extent fused with pronunciation of the word roots. Therefore positions of laryngeal muscles for pronouncing word roots should be concordant with pronouncing affixes. Sounds of affixes control to some extent sounds of roots. Affixes are “tale that wag the dog,” like anchors keeping the word sounds, and therefore historical emotions. Languages with many affixes tend to keep their sounds changes slow.

For example, Middle English, similar to other Germanic languages, had a number of inflections. About 500 years ago during transition to Modern English most of inflections have been lost (remaining inflections include “ed” for the past tense and “s” for plurals). English lost anchors for its sounds, and sounds of English started changing fast (Lerer, 2007). English sounds significantly change in each generation. e.g., a well know change is the Great Vowel Shift. Much less research have been devoted to losing historical connections between word semantic meanings and corresponding emotions. This has led to low emotionality of contemporary English.

This low emotionality makes English a powerful tool of semantic thinking. Ancient emotions determined by language sounds and unrelated to semantic contents do not interfere with the thought train. English is very good for science and engineering (Perlovsky, 2013c, 2016a). The other side of low emotionality is that English is losing historical connections of value words to cultural values evolving over millennia. Recent generations change cultural values according to current fads; a lot of people think that this is possible because today people are smarter than in the old days, and therefore are not bound by meaningless traditions. It is not appreciated that this freedom from traditional values (good or bad) is due to the fact that English language sound are changing fast and for this reason English is literally “losing anchors,” which is not a guarantee that current fads are better than millennial traditions.

On the “other side” of language emotionality is Arabic language. It is a fusional language, in which inflections are strongly fused with word roots. It follows that sounds of Arabic change slowly (if at all). Semantic meanings of Arabic words are strongly connected to historically ancient emotions. Arabic may not be flexible for scientific thinking. But Arabic moral values are strongly rooted in history. Many Arabic people therefore are sure about their moral values. It is important to appreciate that current contradictions between Arabic and English speaking cultures do not depend on specific political leaders, but are rooted in the very sounds of Arabic and English languages (Perlovsky, 2009b, 2011a, 2012d).

Again, this section gives an example of a vast field of complicated cognitive and language mechanisms as well as their affects on cultures. A vast field of knowledge is explained from the first principles; a theory makes predictions that can be tested in experiments; few of these predictions have been tested experimentally, these are directions for future research. It answers many questions that could not even have been formulated previously. Mathematical models are discussed in given references. The theory discussed in this section is a part of physics of the mind, it is a step toward making psychology a “hard” science.

## Cognitive functions of music

Cognitive functions of music, the reasons for its evolution from pre-human vocalizations to Beethoven, Chopin, and Justin Bieber could not have been understood. Aristotle (1995) asked “why music being just sounds reminds the states of soul?” Kant could not understand the role of music in cognition Kant (1790). Darwin (1871) thought that music is the “greatest mystery.” And contemporary musicologists could not find an answer (Editorial, 2008; Honing et al., 2015).

An explanation of music cognitive functions have been derived from the dual model (Perlovsky, 2006a, 2010a, 2012b,c, 2013a, 2014, 2015a,b). Evolution of language led to explosion of knowledge and a number of concepts. Concepts contradict other concepts to some extent. These contradictions among concepts dissatisfy the knowledge instinct and produce unpleasant emotions, cognitive dissonances (Festinger, 1957). Cognitive dissonances are immediately resolved: the new contradictory knowledge is discarded fast and usually without reaching consciousness (Jarcho et al., 2011). It follows that evolution of language, cognition, and culture required a cognitive mechanism for overcoming cognitive dissonances without rejecting knowledge.

This mechanism had to act fast and to be related to language. And this mechanism existed since the beginning of language evolution, it is language prosody. Low-emotional prosody is overcoming minute cognitive dissonances present in everyday choices. Overcoming stronger cognitive dissonances, which appeared with evolution of language and culture, dissonances related to unrequited love, betrayals by friends and loved one, required stronger emotions. These stronger emotions appeared in songs, an ability which eventually evolved into music (Perlovsky, 2006c, 2016a,b,c).

This theory which relates music evolution to cognitive dissonances explains why many people enjoy listening to sad music. Some music is so sad it cannot be listened without tears. Listeners of the BBC's Today program in 2004 voted Barber's Adagio for Strings the “saddest classical” work ever. It is among the highest-selling classical music piece. The physical theory of the mind described here explains a mysterious power of music over us as well as Biblical statement: “in much wisdom is much grief” (Ecc. 1:18). For we leave in the sea of cognitive dissonances, in the sea of grief. Music helps us overcome the grief of knowledge and to continue developing the culture.

The theoretical prediction, resolving the millennial mystery of music by relating it to cognitive dissonances have been confirmed experimentally (Masataka and Perlovsky, 2012a,b, 2013; Cabanac et al., 2013; Perlovsky et al., 2013). This theory opens a vast field for future research, including experimental measurements of musical emotion, e.g., what is the emotional distance between a musical phrase from Beethoven and another musical phrase from Chopin. How many musical emotions exist?

## Dynamic logic, DL

DL is a mathematical technique modeling the knowledge instinct, or more specifically, the brain-mind mechanism of matching vague top-down signals to bottom-up signals without computational complexity (Perlovsky and McManus, 1991; Perlovsky, 2001, 2006a,b; Perlovsky et al., 2011; Vityaev et al., 2011; Kovalerchuk et al., 2012; Perlovsky and Shevchenko, 2014). It is a mathematical foundation of the physics of the mind and all results discussed in this paper. It is a fundamental principle of the mind describing the process from vague to crisp representations.

The mathematical description, following (Perlovsky et al., 2011) is given below. An index m numbers top representations; an index n numbers bottom representations; an index i numbers BU signals making up the n-th representation. Parameters x_ni_ measure the strength of association of the BU signal i with bottom representation n, and p_mi_ measure the strength of association of the BU signal i with top representation m. Values of these parameter are limited between 0 and 1. Associations between top and bottom representations are modeled by

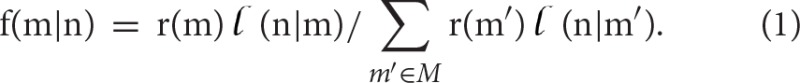

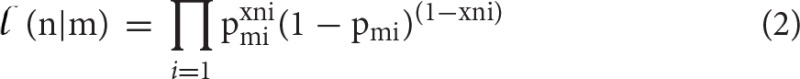





Here 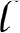 (n|m) are pdf-like measures, and f(m|n) are probabilities-like measures, similar to a posteriori Bayes probabilities. Under certain conditions, these variables indeed can be interpreted as probabilistic measures. For preserving these probabilistic interpretations 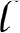 (n|m) is defined so that integration over x yields 1. And parameters r(m) are used to model the proportion of signals m in top-down representations. These representations model a single level in the hierarchical mental structure; at the lowest level of the hierarchy x_ni_ represent sensor signals: if a feature i is present in object or event n, x_ni_ = 1, otherwise 0.

Learning in DL processes constitutes adapting parameters p_mi_ and r(m) so that top representations m correspond to patterns in bottom representations x_ni_. This process maximizes a total similarity measure between all bottom patterns and top representations,

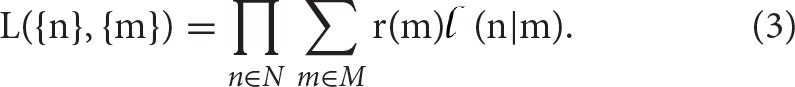



Maximizing this similarity is a model of KI.

The learning process maximizing KI (Perlovsky et al., 2011) can be specified iteratively,

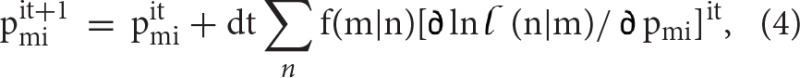

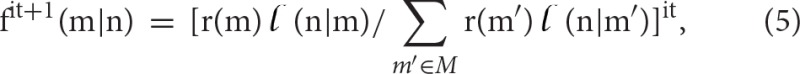





(6) $${\text{r}}^{\text{it}+1}(\text{m})=[(\text{1/N})/\sum_{n}\text{f}(\text{m|n}){]}^{\text{it}},$$

In equation (4) a parameter dt is an increment of the internal time t of the DL iterations. A fundamental aspect of the DL learning is an initial vague state, which is achieved by specifying the unknown parameter values p_mi_ near 0.5. This value of p_mi_ corresponds to maximal variances of 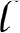 (n|m) and vague representations f(m|n). This state corresponds to the Aristotelian potentiality. In the process of perception, “mind meets matter,” TD and BU signals interact and representations reach crisp states corresponding to the Aristotelian actualities. We show in an example below that this process converges fast.

**Figure 3:**
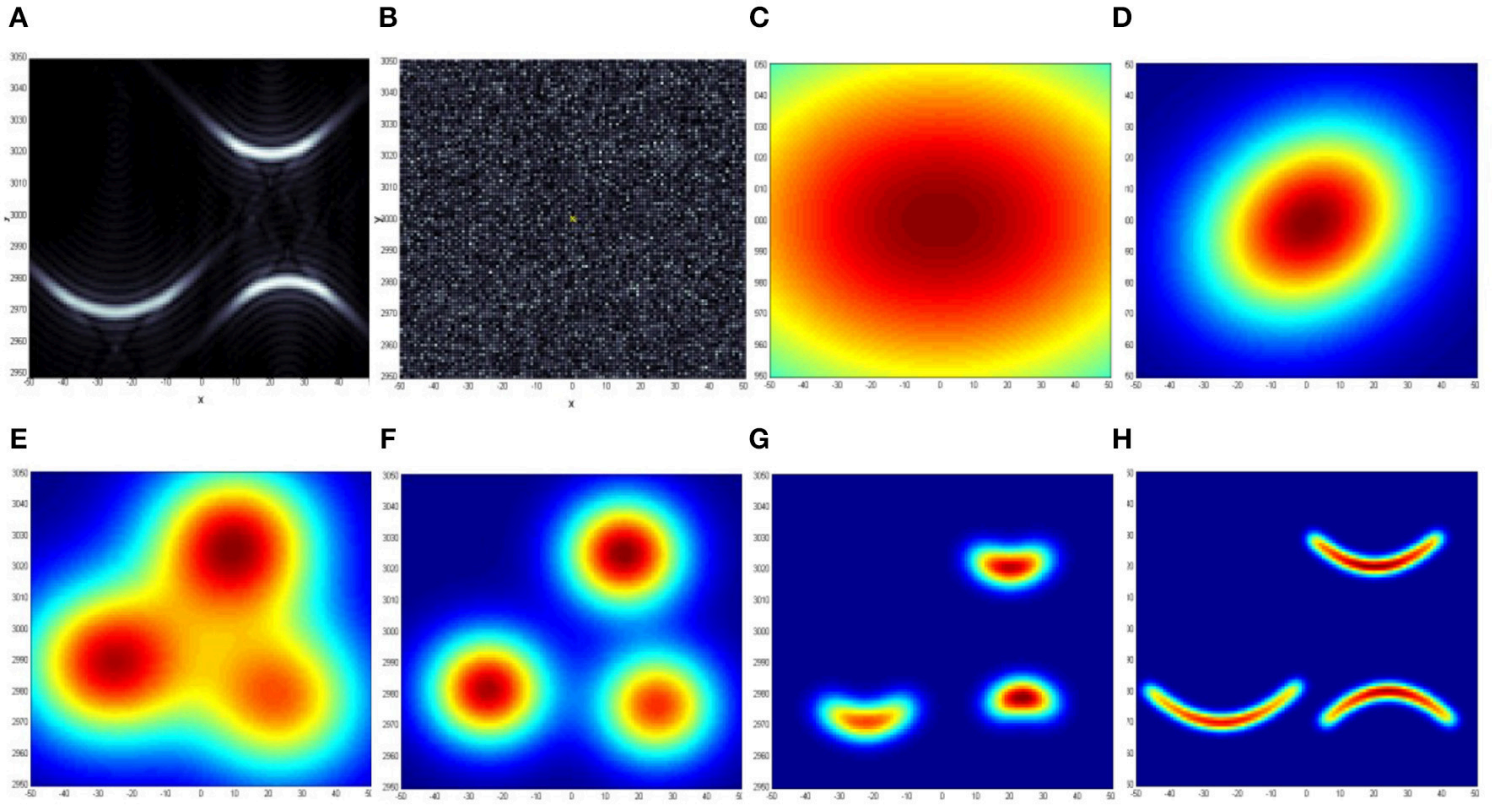
**Perception of “smile” and “frown” patterns in noise, an example of dynamic logic “from vague-to-crisp” process: (A)** true “smile” and “frown” patterns are shown without noise; **(B)** actual image available for recognition (signals are below noise, signal-to-noise ratio is about 1/3); **(C)** an initial vague concept-model; **(D)** through **(H)** show improved concept-models at various iteration stages (total of 21 iterations). Between stages **(D)** and **(E)** DL tries to fit the data with more than one model and decided, that it needs three models to “understand” the content of the data. Until stage **(G)** the DL “thought” in terms of simple blob models, at **(G)** and beyond, the algorithm decided that it needs more complex parabolic models to describe the data. Iterations stopped at **(H)**, when similarity (3) stopped increasing.

In this example, Figure 3. illustrates the DL perception of “smile” and “frown” patterns in noise. Patterns without noise are shown in A; with noise, as actually measured they are shown in B.

When models come close to the true shape, iteration 17, Figure 3G, there is sufficient sensitivity to determine that parabolic shapes better match signals, three parabolic shapes are activated. At iteration 21, Figure 3H, iterations stop, because similarity (3) stopped increasing with iterations. The number of computer operations in this example was about 10^9^. Thus, a problem that was not solvable due to CC becomes solvable using DL.

To summarize this example, during DL learning initial vague and uncertain models (Aristotelian potentialities) are associated with structures in the input signals (Aristotelian forms interact with matter), and vague models become more definite and crisp with successive iterations. In the image available for recognition, Figure 3B, signal is below noise, signal-to-noise ratio is about 0.3. This is a significant improvement over other state-of-the-art practically working algorithms; a standard required signal-to-noise ratio is more than 30. The achieved improvement is about 100 times.

The above formulation of DL includes dual models as well as the dual hierarchy. Some x_ni_ and p_mi_ correspond to cognitive representations and other correspond to language representations. Language representations exist in surrounding language and are learned early in life. Cognitive representations are learned from experience under guidance of existing language representations. Existing preliminary simulations of systems with cognitive and language data indicate that interactions of cognition and language can self-organize by association of both types of representations in a single model. This process could be speeded up if certain associations among vague models are inborn. Understanding inborn associations is a future research direction.

Adequacy of DL for modeling neural mechanisms of perception has been experimentally proven in (Bar et al., 2006; Kveraga et al., 2007).

## Conclusion

This paper establishes a new area of science, physics of the mind. Physics of the mind, let's repeat is methodologically similar to all areas of physics in identifying few fundamental principles and their mathematical models, a general mathematical model built from these few principles, describing a vast area of knowledge, and making experimentally testable predictions. Experimental tests of these predictions confirm or disconfirm the theory.

Physicists know that the very first test of a scientific theory is its elegance and beauty; these include Einstein (see McAllister, 1999), Poincare (2001), Dirac (1982). The beauty of a scientific theory is its ability to describe a vast area of knowledge from few basic principles, and to make experimentally testable predictions. The actual experimental tests are the final proof of the theory. Currently a number of theoretical prediction have been experimentally confirmed, even so they are unexpected and go against accepted views.

Still a number of predictions remain to be confirmed, a vast area of theoretical and experimental development is opened for future research. Traditional psychology is a “soft” science that does not develop mathematical models of the mind self-organization based on few principles, describing vast areas of knowledge, and making experimentally verifiable predictions. A new area of science physics of the mind extends psychology toward “hard” sciences.

Opportunities for unified areas of research arise in place of former misunderstandings and contradictions.

## Author contributions

The author confirms being the sole contributor of this work and approved it for publication.

### Conflict of interest statement

The author declares that the research was conducted in the absence of any commercial or financial relationships that could be construed as a potential conflict of interest.
